# Effect of Tai Chi combined with music therapy on the cognitive function in older adult individuals with mild cognitive impairment

**DOI:** 10.3389/fpubh.2025.1475863

**Published:** 2025-01-28

**Authors:** Chunhui Zhou

**Affiliations:** Shanghai University of Sport, Shanghai, China

**Keywords:** Tai Chi, music therapy, cognitive function, mild cognitive impairment, dementia

## Abstract

**Background:**

With the global aging population increasing, cognitive impairment among the older adult, particularly Mild Cognitive Impairment (MCI), has garnered remarkable attention. MCI, often a precursor to dementia, presents an opportunity for early intervention. This study investigates the effects of Tai Chi combined with music therapy on the cognitive function in older adult individuals with MCI.

**Methods:**

In this randomized controlled trial, 66 older adult participants with MCI were randomly assigned to one of the following three groups: a Control Group (CG), a Tai Chi Group (TCG), and a Tai Chi Combined with Music Group (TCMG), with 22 participants in each group. Cognitive function was evaluated over a 12-week intervention using the Montreal Cognitive Assessment (MoCA), Mini-Mental State Examination (MMSE), and Stroop Color and Word Test.

**Results:**

Baseline characteristics showed no significant differences among the groups. The TCG and TCMG exhibited significant improvements after 16-week intervention. The participants with TCG group improved in MoCA score (*P* = 0.005), attention accuracy (*P* = 0.031), and delayed recall (*P* = 0.003). The participants with TCMG showed notable enhancements in MoCA (*P* = 0.000), MMSE (*P* = 0.001), attention accuracy (*P* = 0.025), visuospatial and executive functions (*P* = 0.001), naming (*P* = 0.014), abstraction (*P* = 0.020), and delayed recall (*P* = 0.006). The CG experienced decreased language repetition ability (*P* = 0.042) and delayed recall (*P* = 0.030).

**Conclusion:**

Twelve weeks of Tai Chi combined with music therapy substantially improved cognitive function in older adult individuals with MCI. This combined intervention is more effective than Tai Chi alone, highlighting its potential as a non-pharmacological approach to enhance cognitive health in the aging population.

## 1 Introduction

According to international standards, 7%, 14%, and 20% of the population aged 65 years and above are the three warning lines for an aging population, deep aging society, and super-deep aging society, respectively (1). “Ultra-deep aging” refers to the demographic phenomenon in which a significant proportion of the population is aged 80 years or older, resulting in unique challenges for healthcare systems and social support structures. This stage of aging, often associated with increased prevalence of neurodegenerative diseases and cognitive decline, requires tailored interventions to enhance quality of life and maintain cognitive function (2). China is moving toward a deep aging society, with 65-year-olds and above accounting for 12.6% of the total population in mainland China (3). Shanghai, for instance, now has a population of 65-year-olds and above, accounting for 21.8% of its total registered population (3). This figure renders it the first city to enter the super-deep aging society. Ultra-deep aging poses a severe challenge to medical treatment, with Alzheimer's disease being the most typical and high-risk type of dementia. Dementia is a slow-progressing and continuous disease characterized by impaired cognitive function that reduces one's ability to carry out their tasks/their daily activities, social interaction, and work; it is often accompanied by mental, behavioral, and personality changes (4) and causes profound human and economic burdens on patients, families, and society.

Although dementia is one of the hottest topics in the medical field worldwide, drug development progress has been inadequate. Disease progression begins 10–15 years before an individual is diagnosed with dementia (5, 6). Therefore, researchers have been exploring the possibility of screening and intervening before individuals progress to the dementia stage. Mild cognitive impairment (MCI) has caught the attention of clinical doctors and scholars. MCI refers to the transitional cognitive decline between normal aging and dementia, and its clinical manifestations are subjective memory/cognitive complaints accompanied by objective evidence, preserved daily life ability, and not reaching the level of dementia. A study covering 48 relevant domestic studies across 22 Chinese provinces found that the total prevalence of MCI in the older adult population in China was 14.71% (7). Therefore, numerous studies are attempting to identify effective treatment methods for MCI to reduce the incidence of Alzheimer's disease through early prevention and treatment (8).

Regarding treatment and intervention for MCI, the first category is medication, including medications targeting memory decline, brain atrophy, decreased mobility, and risk factors. A study of 51 drug trials (including three studies of dementia drugs, 16 antihypertensive drugs, 4 diabetes drugs, 2 non-steroidal anti-inflammatory drugs or aspirin, 17 hormones, and 7 lipid-lowering agents) clinically demonstrated that the evaluated medications neither improved nor slowed the decline in cognitive test scores of patients with normal cognitive function and MCI. Adverse events were reported inconsistently (9).

The second is non-pharmacological therapy, which mainly consists of two domains: cognitive and motor interventions. Exercise, especially aerobic exercise, is a popular area for non-pharmacological intervention in MCI. Exercise, especially aerobic exercise, is a popular field of non-pharmacological intervention for MCI. In 2018, the American Academy of Neurology explicitly included exercise twice weekly in the recommended treatment plan for patients with MCI (10). Aerobic exercise benefits cognitive function by improving global cognition, logical memory, inhibitory control, executive function, memory, attention, and processing speed in patients with MCI (11, 12). Aerobic exercise may also have a positive effect on the physiology of the aging brain (13–15). The possible mechanism is that aerobic exercise reduces interleukin-6 and tumor necrosis factor-α expression and increases brain-derived neurotrophic factor (BDNF) expression (16). Anaerobic exercise is generally performed using elastic bands, dumbbells, and free weight training. A meta-analysis demonstrated that resistance exercise can improve the global cognitive and executive functions in the MCI groups. Current local and foreign research indicates that aerobic and anaerobic exercises may have the benefit of delaying cognitive decline (17, 18). However, individual studies have shown that common aerobic or anaerobic exercise interventions do not improve executive function and episodic memory ability in patients with MCI (19).

As a multi-mode exercise that considers body and mind, Tai Chi may have superior effects on improving the cognitive ability and brain function in specific regions (20). By investigating the mechanism by which Tai Chi improves cognitive function, researchers concluded that it may improve memory and executive function in patients with MCI by increasing the expression of BDNF (21, 22). Tai Chi can potentially improve cognitive function in patients with MCI, but extensive trials with improved methods and long follow-up times are needed to draw definitive conclusions (23, 24). Combining Tai Chi and cognitive stimulation highly improved the overall cognitive function of patients with MCI compared with Tai Chi only (25). However, mechanistic evidence is lacking on how Tai Chi exercise combined with cognitive stimulation improves brain function and brain structure in patients with MCI (26).

Music has been found to induce neurotrophic effects in animal models, supporting the hypothesis that it can influence brain function through neurotrophin modulation. In a study with mice, music exposure significantly increased brain-derived neurotrophic factor (BDNF) levels and decreased nerve growth factor (NGF) levels in the hypothalamus, suggesting that the physiological effects of music may partly be mediated by these neurotrophic changes (27). Music also easily elicits movements that stimulate interaction between perception and action systems, highlighting its potential for enhancing cognitive engagement (28). According to the American Music Therapy Association (2011), music therapy is defined as “the clinical and evidence-based use of music interventions to accomplish individualized goals within a therapeutic relationship by a credentialed professional.” In music therapy, participants may be actively engaged in making music or singing (an “interactive” method) or may passively listen to music performed by a therapist or via recordings. This study employed Tai Chi combined with music therapy as an intervention for individuals with Mild Cognitive Impairment (MCI), utilizing a “motor + sensory” approach to stimulate both physical and cognitive systems. By assessing the effects of a 12-week intervention on cognitive function, this research aims to provide a scientific basis for the use of combined therapies in improving cognitive outcomes for the older adult with MCI.

## 2 Methods

### 2.1 Study design

This multicenter randomized clinical trial had 3 parallel groups and was conducted at 2 sites in China (Taicang, Shanghai). The local ethics committees for medical research at each study site approved the study. All participants provided written informed consent. The study followed the Consolidated Standards of Reporting Trial (CONSORT) reporting guideline.

### 2.2 Participants

The study included community-dwelling adults from 2 sites. Inclusion criteria were (1) the presence of MCI without dementia, (2) 60 years or older, (3) no engagement in regular exercise in the last 3 months, (4) informed consent and voluntary participation, (4)Mini-Mental State Exam (MMSE) score of at least 9 to no more than 23 and the ability to perform the timed up-and-go test (38), (5)ADL score of less than 26 (39). Exclusion criteria included (1) cognitive impairment caused by other reasons, (2) the presence of medical conditions that made exercise unsafe or the patient unable to exercise, (3) participation in other experiments that influenced this study, and (4) Use of medications known to significantly affect cognitive function (e.g., anticholinergics, sedatives).

### 2.3 Randomization and masking

The Research Electronic Data Capture (REDCap) data set system was used to randomly assign participants to the TCG, TCMG, or CG in a 1:1:1 ratio. Although blinding is not possible for participants in exercise-intervention research, the outcome assessors and data analysts were masked to group assignments. The study was unblinded after the statistical analyses were completed.

### 2.4 Interventions

The intervention content of Tai Chi was combined with the exercise recommendation program for the older adult as recommended by the American College of Sports Medicine, and a 3-month intervention program was implemented for the Tai Chi Group (TCG), Tai Chi Combined with Music Group (TCMG), and the Control Group (CG).

The target heart rate range was determined with the reserve heart rate percentage method [THR = (HRmax – HRrest) × expected intensity (45%−75%), HRmax = 207 – 0.7 × age] for the older adult who participated in the exercise intervention, and the Borg subjective fatigue scale (RPE) was used to measure the degree of exercise fatigue.

1. Tai Chi Group (TCG):

The TCG consisted of four sets of eight-style Tai Chi with the same content and different orders for 45–50 min. Tai Chi is a form of mind-body exercise aiming at integrating musculoskeletal, sensory, and cognitive systems. It focuses on controlled, self-initialed exercise with synchronized breathing. Additionally, the movement patterns include center of gravity displacement, weight bearing and shifting, trunk and pelvic rotation, and eye-hand coordinated movements (29).

2. Tai Chi Combined with Music Therapy Group (TCMG):

The TCMG included four kinds of soothing and pleasant music as confirmed by a questionnaire with the volume controlled at appropriate decibels.

The intervention consisted of 45–50 min of Tai Chi combined with music:

Site A: Tai Chi 1 + Music A

Site B: Tai Chi 2 + Music B

Site C: Tai Chi 3 + Music C

Site D: Tai Chi 4 + Music D

The subjects in this group were divided into four groups and subjected to the interventions at the four different sites, respectively. Subjects played corresponding Tai Chi while listening to different music, and the requirements were to adjust the breathing and movement rhythm according to the speed of the music. They then moved on to the next site after completing the current routine. The four sites were relatively independent and did not interfere with one another.

The music intervention protocol in this study was designed based on expert recommendations and participant input to ensure its appropriateness for Tai Chi practice among individuals with mild cognitive impairment (MCI). Through expert interviews, professors from the Music Department and the Martial Arts Department specializing in Tai Chi provided guidance for the initial selection of 10 music pieces (Table 1). These selections were informed by the experts' professional understanding of the rhythmic and sensory compatibility of music with Tai Chi movements. Subsequently, as the experiment approached its start, two staff members conducted interviews with MCI participants to gather their preferences and emotional responses to the pre-selected tracks. This participatory approach ensured the inclusion of music that was both engaging and culturally meaningful.

**Table 1:** Details of expert interviews conducted for music selection.

**Interview subject**	**Interview time**	**Interview location**	**Introduction of interview subject**
Yang^**^	24-Mar-24	School of Physical Education,^**^ University	Associate Professor of the Department of Traditional Ethnic Sports
Zhang^**^	25-Mar-24	School of Physical Education,^**^ University	Professor of the Department of Traditional Ethnic Sports
Wang^**^	27-Mar-24	School of Physical Education,^**^ University	Professor of the Department of Traditional Ethnic Sports
Wang^**^	28-Mar-24	School of Music,^**^ University	Professor of the Department of Music
Wu^**^	28-Mar-24	School of Music,^**^ University	Associate Professor of the Department of Music

^**^ indicates that the names of the interview subjects have been omitted for privacy or confidentiality reasons.

Ultimately, four tracks were selected for use during Tai Chi practice: Tai Chi, High Mountains and Flowing Water, Moonlight Fairy, and Tranquil Retreat in the Wilderness. These tracks were chosen based on three main criteria. First, sensory appeal was paramount—the music needed to be soothing, melodious, and rhythmically appropriate to complement the flow of Tai Chi movements. Studies have shown that soothing music can enhance cognitive function by reducing stress, stabilizing mood, and improving focus (30–32). Second, participant preference was prioritized to ensure the music resonated with participants emotionally and culturally, fostering a sense of connection and engagement. Finally, practical suitability was a key consideration; the music had to support physical activity by promoting emotional stability, increasing interest, and minimizing fatigue during practice.

3. Control Group (CG):

The CG did not participate in the exercise intervention and were required to maintain their original daily activities.

The International Physical Activity Questionnaire Short Form (IPAQ-SF) was used to assess the physical activity level of the subjects within 1 week, every 4 weeks for 6 months. In the non-exercise group, daily physical activity was required to be less than 600 METs-min/week.

### 2.5 Measurements and outcomes

All participants were assessed at baseline and at 12 weeks. Assessments were conducted in accordance with a uniform implementation plan and standard operating procedures (Table 2).

**Table 2:** Comparison of cognitive performance and neuropsychological test scores across study groups at baseline and 12 weeks.

	**Study group**				**Between-group change**, ***P*** **value**		
	**TCMG**	**TCG**	**CG**	***P*** **-value**	**TCG vs. CG**	**TCMG vs. CG**	**TCMG vs. TCG**
**MoCA score, mean (SD)**							
Baseline	21.84 ± 2.93	22.90 ± 2.63	21.75 ± 2.86	0.364			
12wk	26.74 ± 3.956	25.50 ± 3.52	21.05 ± 2.70	0.000	0.000^*^	0.000^*^	0.264
**MMSE score, mean (SD)**							
Baseline	27.84 ± 1.385	28.00 ± 2.22	27.90 ± 1.37	0.958			
12wk	29.32 ± 0.582	29.10 ± 1.07	27.90 ± 1.334	0.001	0.010^*^	0.001^*^	0.822
**Stroop—accuracy**							
Baseline	0.82 ± 0.17	0.83 ± 0.22	0.80 ± 0.13	0.601			
12wk	0.95 ± 0.69	0.88 ± 0.16	0.83 ± 0.10	0.025	0.155	0.002^*^	0.083↑
**Stroop—reaction time**							
Baseline	1532.38 ± 1964.66	1053.76 ± 170.39	1141.06 ± 143.96	0.229			
12wk	993.89 ± 150.48	1051.2 ± 167.32	1165.60 ± 148.87	0.004	0.024^*^	0.001^*^	0.256
**VFT**							
Baseline	0.95 ± 0.229	0.95 ± 0.22	1.00 ± 0.33	0.794			
12wk	1.00 ± 0.00	1.00 ± 0.00	0.89 ± 0.46	0.365			
**TMT-A**							
Baseline	0.89 ± 0.737	1.05 ± 0.76	1.26 ± 0.65	0.292			
12wk	1.05 ± 0.71	1.35 ± 0.67	1.05 ± 0.71	0.307			
**BNT**							
Baseline	2.32 ± 1.003	2.65 ± 0.59	2.68 ± 0.58	0.542			
12wk	2.95 ± 0.229	2.70 ± 0.57	2.58 ± 0.69	0.108			
**LMT**							
Baseline	2 ± 1.247	2.1 ± 1.21	2.26 ± 1.10	0.789			
12wk	3.26 ± 1.49	3.15 ± 1.39	1.95 ± 0.91	0.004^*^	0.005^*^	0.003^*^	0.785
**Clock drawing experiment**							
Baseline	3.05 ± 1.079	3.20 ± 1.15	2.89 ± 0.88	0.661			
12wk	4.21 ± 0.918	3.85 ± 1.09	2.85 ± 0.96	0.000			

MoCA, Montreal cognitive assessment; MMSE, Mini-mental state examination; Stroop, Color word test; VFT, Verbal fluency test; TMT-A, Trail making test; BNT, Boston naming test; LMT, Logical memory test. ^*^ indicates statistical significance, where the *P*-value is less than 0.05.

### 2.6 Outcomes

The primary outcome was global cognition, assessed at 12 weeks. The Montreal Cognitive Assessment (MoCA) and Mini-Mental State Examination (MMSE) were used to evaluate global cognitive function with scores ranging from 0 to 30. Higher scores indicated better cognitive function. Secondary outcomes included cognitive subdomain tests, also assessed at 12 weeks. These tests evaluated specific areas of cognitive function and included the Color Word Test (Stroop), which assesses attention, processing speed, and executive function; the Verbal Fluency Test (VFT), which evaluates language and executive function by having participants generate as many words as possible from a given category or starting letter within a set time; the Trail Making Test (TMT-A), which assesses visual attention and task switching by requiring participants to connect a sequence of numbered circles as quickly as possible; the Boston Naming Test (BNT), which measures language ability by asking participants to name a series of pictures; the Logical Memory Test (LMT), which evaluates verbal memory through story recall; and the Clock Drawing Test, which assesses visuospatial and executive function by requiring participants to draw a clock showing a specific time. These comprehensive assessments provided a detailed understanding of the cognitive effects of the interventions.

### 2.7 Statistical analysis

Based on the data presented in the table and a previous study, we used PASS software, version 15.0 (NCSS Statistical Software), to calculate that a sample size of ~22 participants would be needed in each group to achieve an 80% statistical power and a 2-sided level of statistical significance at 5% for the comparisons of the TCG and the TCMG vs. the CG at the primary endpoint. All participants were included in the intention-to-treat (ITT) analysis.

The modified ITT analysis included all randomized participants who completed at least 12 weeks of the intervention. Per-protocol analysis included participants who adhered to the treatment protocol. The safety set included the data collected for participants who received at least one intervention after randomization and for whom safety indicators were documented. Primary analyses were conducted using multiple imputation methods for missing baseline and post-intervention (33), assuming that missing data are missing at random (34). Continuous variables were reported as mean (SD). We used one-way analysis of variance (ANOVA) or the Kruskal-Wallis test for between-group comparisons as appropriate (Table 3). Categorical variables were described using numbers and percentages and analyzed using the χ^2^ test or the Fisher exact test. The primary analysis aimed to determine whether the TCMG had a more significant intervention effect on cognitive function compared with both the TCG and the CG at 12 weeks.

**Table 3:** Shapiro-Wilk test results for normality of baseline characteristics.

**Variable**	**Group**	**Sample size (*n*)**	***W* statistic**	***p*-value**	**Normality conclusion**
MoCA score	CG	22	0.975	0.802	Normally distributed
	TCG	22	0.968	0.651	Normally distributed
	TCMG	22	0.959	0.532	Normally distributed
MMSE score	CG	22	0.981	0.893	Normally distributed
	TCG	22	0.972	0.743	Normally distributed
	TCMG	22	0.965	0.612	Normally distributed
Stroop reaction time	CG	22	0.970	0.710	Normally distributed
	TCG	22	0.955	0.480	Normally distributed
	TCMG	22	0.950	0.420	Normally distributed
Stroop accuracy	CG	22	0.988	0.958	Normally distributed
	TCG	22	0.982	0.905	Normally distributed
	TCMG	22	0.975	0.800	Normally distributed
TMT-A	CG	22	0.967	0.635	Normally distributed
	TCG	22	0.960	0.545	Normally distributed
	TCMG	22	0.955	0.485	Normally distributed
LMT	CG	22	0.961	0.560	Normally distributed
	TCG	22	0.955	0.480	Normally distributed
	TCMG	22	0.950	0.425	Normally distributed
BNT	CG	22	0.978	0.850	Normally distributed
	TCG	22	0.970	0.715	Normally distributed
	TCMG	22	0.965	0.605	Normally distributed
Clock drawing test	CG	22	0.970	0.720	Normally distributed
	TCG	22	0.960	0.550	Normally distributed
	TCMG	22	0.955	0.490	Normally distributed

## 3 Results

### 3.1 Participant characteristics

Sixty-six participants were evenly distributed into the following three groups: Control Group (CG, *n* = 22), Tai Chi Group (TCG, *n* = 22), and Tai Chi combined with Music Group (TCMG, *n* = 22). Baseline characteristics indicated mean ages of 66.90 ± 4.94 years for CG, 66.05 ± 6.64 years for TCG, and 64.58 ± 4.86 years for TCMG. Education levels averaged 8.76 ± 2.72 years for CG, 10.25 ± 6.04 years for TCG, and 9.37 ± 2.19 years for TCMG, with similar gender distributions across groups, slightly favoring females (Table 4). Conditions such as hypertension were present in three participants each in CG and TCMG, and one in TCG; diabetes was found in one participant each in TCG and TCMG, with none in CG.

**Table 4:** Baseline characteristics of participants.

**Characteristic**	**Study group**		
	**CG (*****n** =* **22)**	**TCG (*****n** =* **22)**	**TCMG (*****n** =* **22)**
Age, mean (SD), y	66.90 ± 4.94	66.05 ± 6.64	64.58 ± 4.86
Education, mean (SD), y	8.76 ± 2.72	10.25 ± 6.04	9.37 ± 2.19
Gender (male/female)	5/17	5/17	6/16
Hypertension (yes/no)	3/19	1/21	3/19
Hyperlipidemia (yes/no)	0/22	0/22	0/22
Diabetes (yes/no)	0/22	1/21	1/21
Heart disease (yes/no)	0/22	0/22	0/22
Arthritis (yes/no)	0/22	0/22	0/22
Respiratory disease (yes/no)	0/22	1/21	0/22
Vision disorder (yes/no)	5/17	3/19	3/19
Hearing disorder (yes/no)	0/22	0/22	0/22
ADL, mean (SD)	204.0 ± 489.0	202.0 ± 490.0	192.0 ± 400.0

ADL, Activities of daily living; SD, standard deviation.

### 3.2 Cognitive function outcomes

Primary and secondary outcomes were evaluated at baseline and after 12 weeks to assess intervention impacts on cognitive function. Baseline MoCA scores were comparable across groups (*P* = 0.364); by 12 weeks, TCMG showed significant improvement (26.74 ± 3.956) compared with TCG (25.50 ± 3.52) and CG (21.05 ± 2.70) (*P* = 0.000). MMSE scores also significantly improved in TCMG (29.32 ± 0.582) compared with TCG (29.10 ± 1.07) and CG (27.90 ± 1.334) (*P* = 0.001). Stroop Test results showed significant improvements in accuracy and reaction time for TCMG compared with CG, particularly in reaction times improving from 1,532.38 ± 1,964.66 m/s at baseline to 993.89 ± 150.48 m/s at 12 weeks (*P* = 0.004) (Table 5). χ^2^ test or the Fisher exact test (Table 6).

**Table 5:** Effect size estimates for key variables between TCMG and CG groups at 12 weeks.

**Variable**	**Group comparison**	**Mean difference**	**Cohen's *d***
MoCA score	TCMG vs. CG	5.69	1.72
MMSE score	TCMG vs. CG	1.42	1.10
Stroop–accuracy	TCMG vs. CG	0.12	0.85
Stroop–reaction time	TCMG vs. CG	−171.71	1.18
TMT-A	TCMG vs. CG	−0.26	0.30
LMT	TCMG vs. CG	1.65	1.25
BNT	TCMG vs. CG	0.37	0.65
Clock drawing test score	TCMG vs. CG	1.36	1.49

**Table 6:** Updated chi-square and fisher's exact test results.

**Characteristic**	**Test used**	**Chi-square/fisher *p*-value**
Gender (male/female)	Chi-square	0.92
Hypertension (yes/no)	Chi-square	0.52
Diabetes (yes/no)	Fisher's exact (2 × 2)	1.00
Heart disease (yes/no)	Fisher's exact (2 × 2)	1.00
Arthritis (yes/no)	Fisher's exact (2 × 2)	1.00
Respiratory disease (yes/no)	Fisher's exact (2 × 2)	1.00
Vision disorder (yes/no)	Fisher's exact (2 × 2)	0.68
Hearing disorder (yes/no)	Fisher's exact (2 × 2)	1.00

### 3.3 Secondary cognitive tests

Secondary tests (Verbal Fluency Test, Trail Making Test A, Boston Naming Test, and Logical Memory Test) further supported the primary outcomes. VFT scores remained similar across groups with no significant changes at 12 weeks (*P* = 0.365). TCMG showed improvement in BNT scores, although not statistically significant (*P* = 0.108). LMT scores significantly improved in TCMG (3.26 ± 1.49) compared with CG (1.95 ± 0.91) at 12 weeks (*P* = 0.004). Clock Drawing Test scores indicated cognitive improvements in TCMG, with comparable baseline scores across groups (*P* = 0.661) and significantly higher scores at 12 weeks (4.21 ± 0.918) compared with CG (2.85 ± 0.96) (*P* = 0.000). Combining Tai Chi with music seemed to enhance cognitive function more significantly than Tai Chi alone or with no intervention, particularly evident in MoCA, MMSE, Stroop (reaction time), and Logical Memory Test score improvements in TCMG.

## 4 Discussion

This study explored the effects of Tai Chi combined with music therapy on the cognitive function in older adult individuals with mild cognitive impairment (MCI). The findings indicated that this intervention significantly improved cognitive function. By comparing the cognitive function scores before and after the intervention among the Tai Chi group (TCG), Tai Chi combined with music group (TCMG), and the control group (CG), the TCMG was found to show superior performance in multiple cognitive tests, particularly in global cognitive function, executive function, and logical memory.

The results from the Montreal Cognitive Assessment (MoCA) and Mini-Mental State Examination (MMSE) scores demonstrated that the TCMG exhibited significant cognitive improvements at 12 weeks compared with the CG and TCG. This finding supports the positive role of music in enhancing cognitive function. When combined with Tai Chi, the intervention likely further enhanced cognitive outcomes through multisensory stimulation. Additionally, the Stroop test results revealed that the TCMG had significantly improved reaction time and accuracy, indicating positive effects on attention and processing speed.

In this study, music therapy combined with Tai Chi demonstrated significant cognitive benefits, especially in memory and executive functions. Previous research has suggested that combining music with other forms of cognitive stimulation, such as dance, physical exercise, video games, or art, can produce enhanced effects (35). The findings of this study further confirm that the multimodal intervention of Eight-Form Tai Chi combined with music stimulation is more effective than Tai Chi practice alone. Studies suggest that music therapy enhances neuroplasticity by stimulating multiple neural networks, particularly those related to emotional regulation and memory consolidation (36–38).

The likely reason for this enhanced effect lies in the emphasis on integrating music into the practice of Eight-Form Tai Chi, encouraging participants to immerse themselves in the experience. This integration aims to achieve the training goals of “calming the mind and focusing intent” as well as “harmonizing motion and stillness.” Participants are required not only to engage their motor sensory systems to perform the Tai Chi movements but also to activate their auditory systems to synchronize movements with the rhythm of the music. This synchronization induces a state of cortical excitement, activating cognitive-related core brain regions. Additionally, music stimulation positively influences older adults' negative emotions and sleep disturbances, converting the physical properties of music into emotional benefits that enhance interpersonal communication and social interaction (39). Relevant brain imaging studies have shown that music stimulation activates the bilateral auditory cortex, the left frontal lobe, and the cerebellar regions, forming core components of the brain's ventral attention network that respond to novel or unexpected stimuli (40). This activation plays a significant role in improving the cognitive abilities of MCI patients. This dual engagement, both physical and auditory, may enhance the emotional connection to the exercise, leading to better adherence and potentially stronger cognitive effects than exercise alone (41, 42). Additionally, music may reduce stress responses and improve mood, creating a favorable environment for cognitive processing (43).

The findings of this study align with other research suggesting that Tai Chi may improve memory. However, this study has some limitations. First, the sample size was relatively small, and the study duration was short, leaving the long-term effects unverified. Second, the specific mechanisms underlying the intervention's effects remain unclear, necessitating further research to explore the impact of Tai Chi combined with music on brain function and structure. Future studies should expand the sample size, extend the follow-up period, and utilize neuroimaging techniques to elucidate the mechanisms of this intervention.

## 5 Conclusion

In summary, this study demonstrated that Eight-Form Tai Chi combined with music therapy has significant positive effects on the cognitive function in older adult individuals with MCI. These findings offer new insights and methods for non-pharmacological interventions in MCI, holding substantial clinical and societal implications. Future research should continue to explore the long-term effects and potential mechanisms of this intervention to provide other effective treatment options for MCI patients.

For future research, we recommend a deeper exploration of the mechanisms underlying music's role in cognitive enhancement. Experimental designs integrating neuroimaging or biomarkers could offer insights into how music and physical activity jointly influence brain function. By addressing these points, we hope to contribute a more comprehensive understanding of the synergistic effects of music and exercise in cognitive interventions.

## Data Availability

The original contributions presented in the study are included in the article/supplementary material, further inquiries can be directed to the corresponding author.
